# Soft Tissue Management and Prosthetic Rehabilitation in a Tongue Cancer Patient

**DOI:** 10.1155/2013/475186

**Published:** 2013-11-11

**Authors:** Umberto Romeo, Marco Lollobrigida, Gaspare Palaia, Domenica Laurito, Riccardo Cugnetto, Alberto De Biase

**Affiliations:** Division of Oral Surgery, Department of Oral and Maxillofacial Sciences, Sapienza University of Rome, Via Caserta 6, 00161 Rome, Italy

## Abstract

One major challenge in treating head and neck oncologic patients is to achieve an acceptable recovery of physiologic functions compatible with the complete tumor excision. However, after tumor resection, some patients present a surgically altered anatomy incompatible with prosthetic rehabilitation, unless some soft tissue correction is carried out. The aim of the present study is to describe the overall mandibular prosthetic rehabilitation of a postoncologic patient focusing on the possibility of soft tissue correction as a part of the treatment. A 72-year-old woman, who undergone a hemiglossectomy for squamous cell carcinoma several years before, was referred to our department needing a new prosthesis. The patient presented partial mandibular edentulism, defects in tongue mobility, and a bridge of scar tissue connecting one side of the tongue to the alveolar ridge. A diode laser (980 nm) was used to remove the fibrous scar tissue. After reestablishing a proper vestibular depth and soft tissue morphology, two implants were placed in the interforaminal region of the mandible to support an overdenture.

## 1. Introduction

Technical advances in head and neck cancer reconstructive surgery have led surgeons not to consider the complete ablation of neoplasms alone as the focus of treatment planning but rather inseparably from the possibility to return patients, as close as possible, to their premorbid condition. This means the necessity to preserve or restore several essential functions like speech, mastication and deglutition [1, 2]. Dental rehabilitation is an important aspect of such comprehensive treatments, aiming to replace teeth that are missing as a consequence of tumor resection or that patients have already lost.

Oral implants offer many advantages in treating edentulous patients who undergone cancer resection when prosthesis has to fit to an altered anatomy [3]. In particular, implant supported overdentures were demonstrated to be a predictable solution for edentulous patients with excellent long-term prognosis, assuring better retention and stability than conventional dentures [4]. While there is no doubt for postoncologic patients about the benefits of an implant supported rehabilitation in terms of stability and retention [5], some questions still remain about the opportunity of placing implants in those patients who received radiation therapy following the surgical treatment due to the possibility of soft tissue complications and low rates of osseointegration reported in the literature [6]. However, if the first decades of the modern implant era were characterized by an extremely cautious approach with regard to irradiated patients [7], nowadays, also considering the higher survival rates of cancer patients, oral implants are indicated for selected preirradiated patients too.

Another concern regards the relationship between prosthesis and soft tissue. Although many efforts are undertaken by surgeons to limit the degree of anatomical deformities during surgical resections of neoplasms, minor preprosthetic correction of the soft tissue may be helpful in some cases (like the one here presented) to eliminate unacceptable interferences of residual scar tissue with the prosthesis. The reconstruction of oral cavity defects always represents a difficult challenge depending on disease staging and patient condition [8]. Due to this unique and complex three-dimensional anatomical environment, the outcomes of reconstructive procedures are often a compromise that implies functional morbidity and, furthermore, may limit the possibility of an adequate prosthetic rehabilitation. In this regard, some laser systems may be useful to perform soft tissue surgery as an alternative to conventional techniques [9–12]. 

In this report, the overall mandibular prosthetic rehabilitation of a postoncologic patient is presented.

## 2. Case Presentation

**Figure 1:**
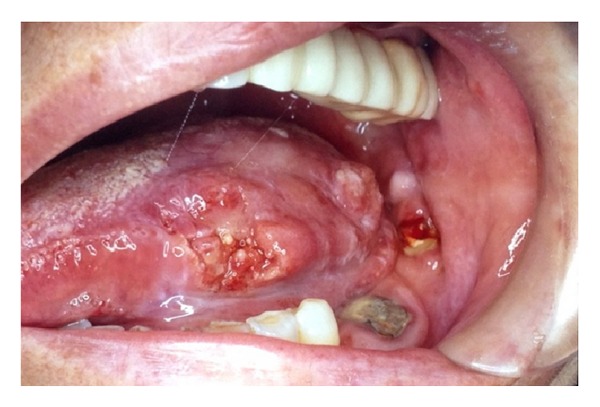
SCC of the left lateral border of the tongue at the time of the primary surgery.

The case refers to a 72-year-old Caucasian woman, diagnosed in 1995 (at the age of 58) with a squamous cell carcinoma (SCC) of the left lateral border of the tongue (Figure 1). The patient underwent hemiglossectomy associated with an en bloc resection of the corresponding lateral floor of the mouth and conservative neck dissection. Postoperative microscopic examination of the specimen revealed a multifocal low grade carcinoma and one lymph node metastasis (pT1pN1pMx G1). The resulting tissue defect was primarily closed with local flaps, and a 6-week postoperative radiotherapy (60 Gy) was delivered. Five months after the primary surgery the patient noted a swelling in the right lateral neck. A submandibular fine-needle aspiration was performed confirming the diagnosis of “metastatic carcinoma.” The patient was then treated with a right conservative neck dissection associated with resection of the submandibular gland. After this second intervention, no signs of recurrent disease were observed, and the patient was rehabilitated with a resin removable partial prosthesis anchored to the remaining teeth. During the following years, the patient lived healthy but lost her teeth due to periodontal disease with the resultant impossibility to wear the prosthesis. 

**Figure 2:**
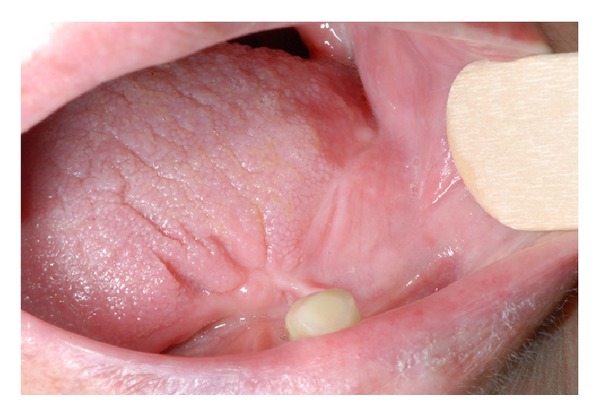
Clinical photograph of the patient's oral cavity as it was presented to the observation. The defect resulting from the hemiglossectomy was primarily closed with local flaps using the left buccal mucosa.

**Figure 3:**
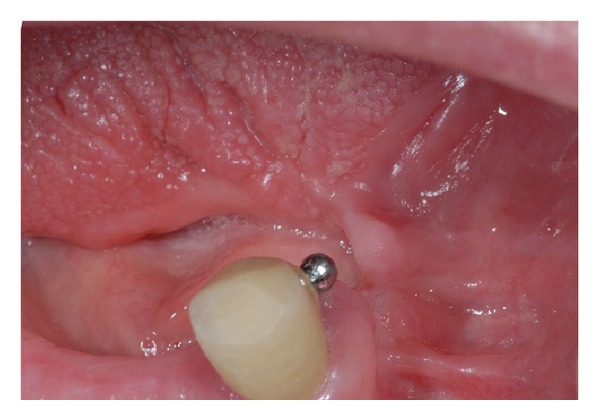
Fibrous scar tissue connecting the tongue to the alveolar ridge mucosa. Scar tissue was responsible for limitation in anterior tongue mobility and speech articulation.

The patient was then referred to our department needing a new prosthesis. At the time of observation, medical history was significant for controlled hypertension and no other systemic diseases. Physical exam revealed defects and limitation in tongue mobility and a bridge of scar tissue connecting one side of the tongue to the alveolar ridge (Figures 2 and 3). The patient was referred for having suffered from limitation in tongue mobility since the surgical excision of the tumour, having difficulties in speech intelligibility and mastication.

After evaluating bone volume with a computed tomography scan and considering patient's complaints about the ability to chew and move the tongue, it was proposed to perform a soft tissue correction before proceeding with the extraction of the only residual tooth and the placement of two endosseous implants to support an overdenture. The aim of the proposed surgical procedure was to reestablish a suitable anatomy eliminating the fibrous scar resulting from the cancer resection, improving tongue mobility, and contextually deepening the sublingual sulcus and vestibular fornix for the receipt of the prosthesis. 

**Figure 4:**
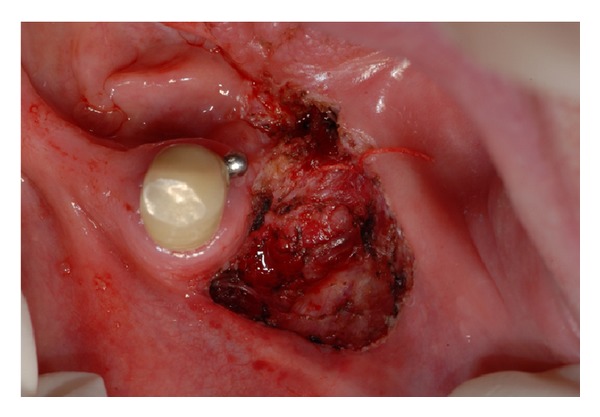
Intraoperatory picture. Tissues were dissected till the mandibular periosteum. Laser thermal effects assured a good visibility during the entire procedure.

**Figure 5:**
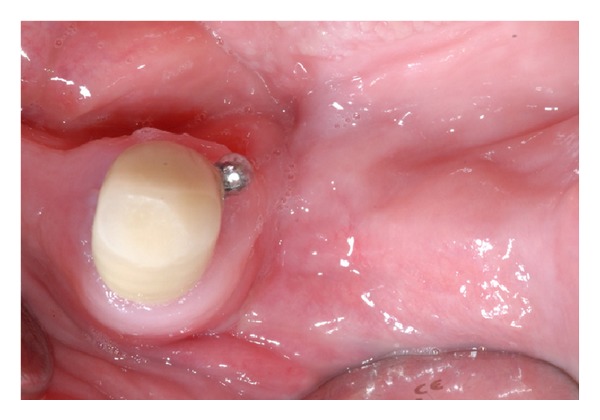
Clinical aspect 28 days after the laser dissection. The wound presented a complete reepithelization, and no scar tissue has formed.

It was used a diode laser (Wiser, Doctor Smile, Brendola, Italy) with a wavelength of 980 nm and 2 W power, operating in continuous-wave mode (CW), an optical fiber of 320 *μ*m, and a fluence of 2488 J/cm^2^. The radiation of this device is selectively adsorbed by hemoglobin, causing a thermal effect that allows a precise surgical cut. After local infiltration of anesthesia (without vasoconstrictor just to enhance hemoglobin light absorption), an incision was made transversally to the ridge involving both the vestibule and the floor of the mouth, dissecting tissues almost till the periosteum and muscles (Figure 4). After simple dissection, no more surgical manipulations were necessary. At the end of the procedure, it was acceptable to allow the laser wound to heal by secondarily epithelialization, and no sutures were applied. The patient was then instructed and informed about the importance of doing tongue exercises (like lifting and protruding) to avoid the formation of new scars. Although no vasoconstrictor had been used, laser-induced coagulation guaranteed an adequate bleeding control during the surgery with good visibility. The procedure was fast and well tolerated. Immediately after the procedure, the patient showed an improvement in both tongue mobility and speech articulation. Gradual reepithelialization and no signs of infection occurred during the following weeks. The patient reported no particular discomfort in the postoperative period, and no scar tissue has formed. At 28 days, the defect was completely closed (Figure 5). 

**Figure 6:**
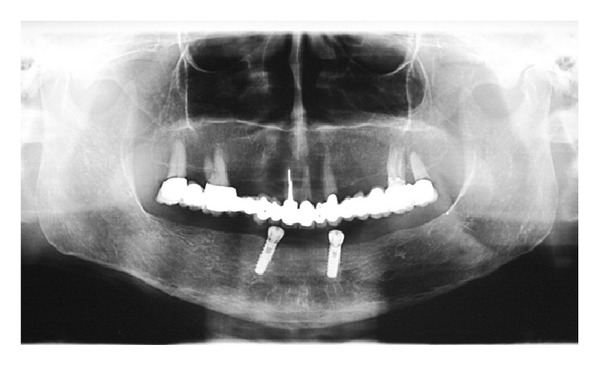
Panoramic radiograph showing the implants placed in the parasymphyseal region of the mandible. Implant supported prosthesis are particularly indicated in postoncologic patients with an altered oral anatomy.

Six months after laser correction and extraction of the residual canine, two implants of 4.1 mm diameter and 13.0 mm length (ExFeel, Megagen Implant Co., Republic of Korea) were placed in the parasymphyseal region of the mandible under the guidance of a surgical template and submerged (Figure 6). At the time of implant placement, primary stability was obtained, and no signs of bone alterations were clinically observed during the healing period.

**Figure 7:**
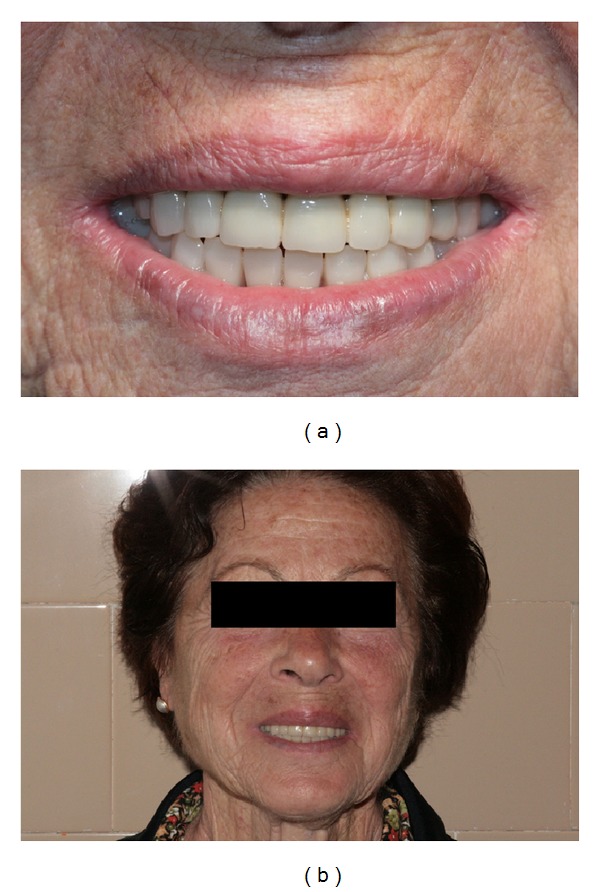
((a)-(b)) Patient's final appearance after the delivery of the prosthesis.

Six months later, an overdenture retained with two free-standing attachments was delivered to the patient. After an early period of adaptation, the patient reported an improved masticatory function in relation to her new diet regimen and defined herself as satisfied (Figures 7(a) and 7(b)).

## 3. Discussion

According to the international literature data, oral cancer represents the eighth most common cancer worldwide, with a constantly higher incidence among men [13]. Oral cancer patients often require a multidisciplinary approach, including surgical resection of the tumor and a series of complementary therapies aiming to a full rehabilitation of the patients. 

Functional rehabilitation represents a fundamental concern in treating head and neck cancer patients, especially when important functions for social life are involved. Missing teeth, which are not replaced with prosthesis, clearly result in a poor quality of life (QOL) for both healthy and oncologic patients. In the latter ones, prosthetic rehabilitation often presents a variety of problems due to the unfavorable anatomy that clinicians may encounter as the result of using various types of flaps to correct large tissue defects of the oral cavity [14]. Implant supported prosthesis may represent a valid solution to overcome such anatomic restrictions. Implant supported overdentures have been proposed as the first choice for treating inferior edentulism [15]. Other authors recommend implant treatment only for those patients who complain about conventional prosthesis [16]. However, it is a fact that patients treated with implant supported overdenture present an improvement in the QOL and, generally, a good level of satisfaction [17, 18]. Furthermore, good long-term results confirm the efficacy and predictability of this prosthetic solution [19]. 

The patient referred to our observation, also after the soft tissue correction, still presented mouth conditions incompatible with a traditional prosthesis (complete denture), as the posterior vestibular depth was totally missing. The absence of any residual tooth and the particular anatomical environment meant that the implant solution was the only possible choice. However, the foremost objective of the treatment was indeed to improve patient's masticatory function and speech articulation, being aware of the impossibility of getting the patient back to her precancer condition. Despite that, the final result was more than acceptable.

Dealing with oncologic patients and dental implants, another important issue that must always be considered is the opportunity of placing implants in those patients who underwent radiotherapy. The suitability of implant placement in irradiated jaws depends on several factors like radiation dose [20], anatomic site [21], and time interval between bone radiation exposure and the implant placement [22]. The main current opinion is that dental implants should be placed in irradiated patients at least 1 year after the radiotherapy [23], offering a stable bone environment to implants [24]. In the case presented, the patient received a radiation therapy a very long time before the secondary implant placement (17 years) and the radiation-related risk of bone complications and/or osseointegration failure was practically absent or, anyway, very low.

Finally, despite the above-mentioned advantages derived from the use of endosseous implants, in some postoncologic patients, it is unavoidable to perform a surgical management of the soft tissue before fabricating an adequate prosthesis. In the case presented, all the well-documented effects of laser surgery were desirable and have been confirmed. The decision to use a diode laser also was derived from the peculiar necessity to cut tissues already manipulated and whose vascularization was unknown by the operators. Taking this into account, laser features were particularly convenient. Fortunately, no hemorrhage or bleeding occurred during the procedure. However, simultaneous cutting and coagulation, allowing an adequate bleeding control and visibility, represent a very useful feature in preprosthetic procedures, particularly in oncologic patients with an altered anatomy. Reduction of postoperative pain and a relatively fast healing process without the necessity of sutures also make laser surgery well accepted by patients. The effects of the soft tissue correction were immediately observable, and the improvement in tongue mobility was clearly noticed by the patient. As result of the soft tissue modification, it was possible to fabricate a suitable inferior overdenture, fully satisfactory for retention, relationship with soft tissue, and aesthetics. 

It is notable that morphology and oral structures arrangement are of primary importance for function and that even little changes in the anatomy may result in significant dysfunctions. This notion guides the reconstructive surgeons in their practice, attempting to limit the degree of permanent impairments resulting from cancer resections. Current multidisciplinary approaches have opened new horizons for cancer patients, aiming not only to eliminate the disease and improve disease-free survival rates but also to “rehabilitate” patients, focusing on an improvement of the QOL [25].

As an integral part of these comprehensive treatments, implant supported prosthesis represent a valid and sometimes the only possible solution to restore mastication and aesthetics in oral cancer patients.
